# Effect of Electrical Impedance Tomography-Guided Early Mobilization in Patients After Major Upper Abdominal Surgery: Protocol for a Prospective Cohort Study

**DOI:** 10.3389/fmed.2021.710463

**Published:** 2021-12-09

**Authors:** Xuan Song, Daqiang Yang, Maopeng Yang, Yahu Bai, Bingxin Qin, Shoucheng Tian, Gangbing Song, Xiuyan Guo, Ranran Dong, Yuanyuan Men, Ziwei Liu, Xinyan Liu, Chunting Wang

**Affiliations:** ^1^Intensive Care Unit (ICU), Liaocheng Cardiac Hospital, Liaocheng, China; ^2^Intensive Care Unit (ICU), Dong E Hospital Affiliated to Shandong First Medical University, Liaocheng, China; ^3^Education Department, Dong E Hospital Affiliated to Shandong First Medical University, Liaocheng, China; ^4^Internal Medicine, Qingdao University, Qingdao, China; ^5^Intensive Care Unit (ICU), Shandong Provincial Hospital Affiliated to Shandong First Medical University, Liaocheng, China

**Keywords:** electrical impedance tomography (EIT), early mobilization, upper abdominal surgery, region of interest, post-operative pulmonary complications

## Abstract

**Background:** Pulmonary complications are common in patients after upper abdominal surgery, resulting in poor clinical outcomes and increased costs of hospitalization. Enhanced Recovery After Surgery Guidelines strongly recommend early mobilization post-operatively; however, the quality of the evidence is poor, and indicators for quantifying the effectiveness of early mobilization are lacking. This study will evaluate the effectiveness of early mobilization in patients undergoing an upper abdominal surgery using electrical impedance tomography (EIT). Specifically, we will use EIT to assess and compare the lung ventilation distribution among various regions of interest (ROI) before and after mobilization in this patient population. Additionally, we will assess the temporal differences in the distribution of ventilation in various ROI during mobilization in an effort to develop personalized activity programs for this patient population.

**Methods:** In this prospective, single-center cohort study, we aim to recruit 50 patients after upper abdominal surgery between July 1, 2021 and June 30, 2022. This study will use EIT to quantify the ventilation distribution among different ROI. On post-operative day 1, the nurses will assist the patient to sit on the chair beside the bed. Patient's heart rate, blood pressure, oxygen saturation, respiratory rate, and ROI 1-4 will be recorded before the mobilization as baseline. These data will be recorded again at 15, 30, 60, 90, and 120 min after mobilization, and the changes in vital signs and ROI 1-4 values at each time point before and after mobilization will be compared.

**Ethics and Dissemination:** The study protocol has been approved by the Institutional Review Board of Liaocheng Cardiac Hospital (2020036). The trial is registered at chictr.org.cn with identifier ChiCTR2100042877, registered on January 31, 2021. The results of the study will be presented at relevant national and international conferences and submitted to international peer-reviewed journals. There are no plans to communicate results specifically to participants. Important protocol modifications, such as changes to eligibility criteria, outcomes, or analyses, will be communicated to all relevant parties (including investigators, Institutional Review Board, trial participants, trial registries, journals, and regulators) as needed via email or in-person communication.

## Background

Post-operative pulmonary complications (PPCs), such as pneumonia, severe atelectasis, and respiratory failure, occur in about 10–50% of patients after a major upper abdominal surgery (1–3). Several factors contribute to the development of PPCs, including post-operative pathophysiological reductions in lung volumes, respiratory muscle function, mucociliary clearance, and pain inhibition of respiratory muscles (4). Following an upper abdominal surgery, PPCs are significantly associated with poor patient outcomes and result in an increased likelihood of rehospitalization, increased mortality and morbidity, longer hospital length of stay, and higher costs (5–10). Enhanced Recovery After Surgery (ERAS) Society Guidelines recommend early mobilization to improve recovery, reduce complication rates, and shorten inpatient hospital stays by reducing the physiologic derangement and stress response after surgery (11–14). While few studies have explored the method and duration for implementing mobilization, the indicators for measuring the effectiveness of post-operative early mobilization on pulmonary outcomes are lacking.

Electrical impedance tomography (EIT) is a non-invasive, radiation-free, bedside monitoring technique that provides dynamic images based on changes in the electrical conductivity of body sections through digital reconstruction (15). Within the chest, impedance can vary dynamically due to changes in lung volume (such as during respiration) and/or due to changes in blood and fluid content (such as within the heart during the cardiac cycle) (16). Thus, EIT can help evaluate the patient's ventilation distribution, which could lead to adjustments to the patient's early mobilization method and duration.

This study aims to evaluate the effect of post-operative early mobilization on lung ventilation function using EIT by assessing the ventilation distribution among various regions of interest (ROI) before and after mobilization in patients undergoing upper abdominal surgery. Additionally, the temporal differences in the distribution of ventilation in various regions of interest during mobilization will be explored to provide a theoretical basis for the development of personalized mobilization plans and precise treatment for patients after upper abdominal surgery.

## Methods and Analysis

### Study Design

This prospective, single-center, single-cohort study will be conducted in the Surgical Intensive Care Unit (SICU) at the Liaocheng Cardiac Hospital in Shandong Province in China.

### Participant Characteristics

Inclusion criteria will consist of the following: patients ≥18 years old admitted to the SICU after undergoing a laparoscopic upper abdominal surgery, such as gastrectomy, pancreaticoduodenectomy, hepatectomy, or esophagectomy, with a written informed consent by the patient or their legally authorized representative. Patients will be excluded for the following reasons: severe pain (11-point numerical rating scale [NRS] ≥7), agitation, ongoing need for ventilation, major hemodynamic instability, cardiorespiratory failure, absence of consent, and technical limitations for EIT monitoring [for example, cardiac electric device, morbid obesity (body mass index [BMI]>30kg/m^2^), or chest bandages (17)]. Patients will also be excluded if mobilization is deemed unsafe for other reasons. Once the patient's eligibility is confirmed, a clinical coordinator will describe each relevant aspect of the study to the patient and obtain a written informed consent from the patient or their legally authorized representative.

### Electrical Impedance Tomography Technique and Measurements

**Figure 1:**
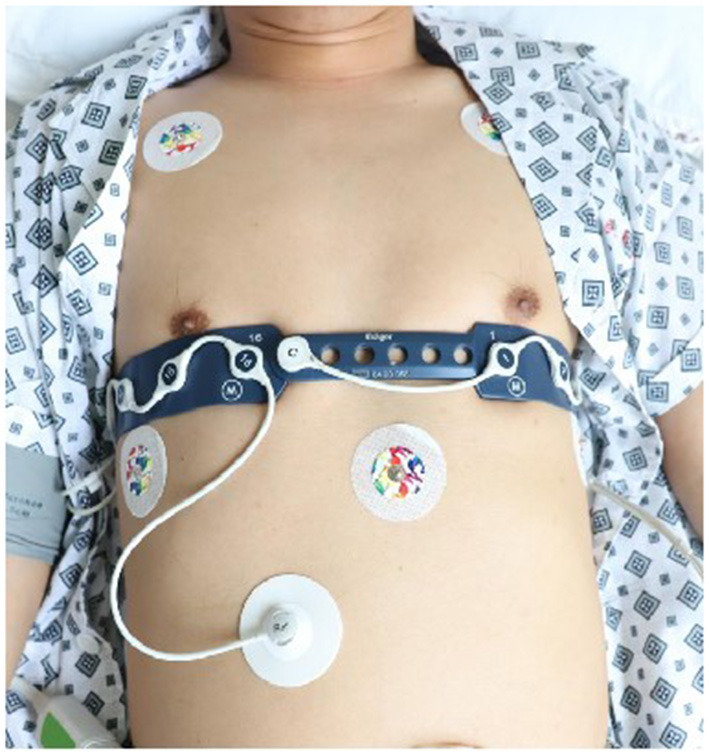
Location of the EIT bind. EIT, electrical impedance tomography.

**Figure 2:**
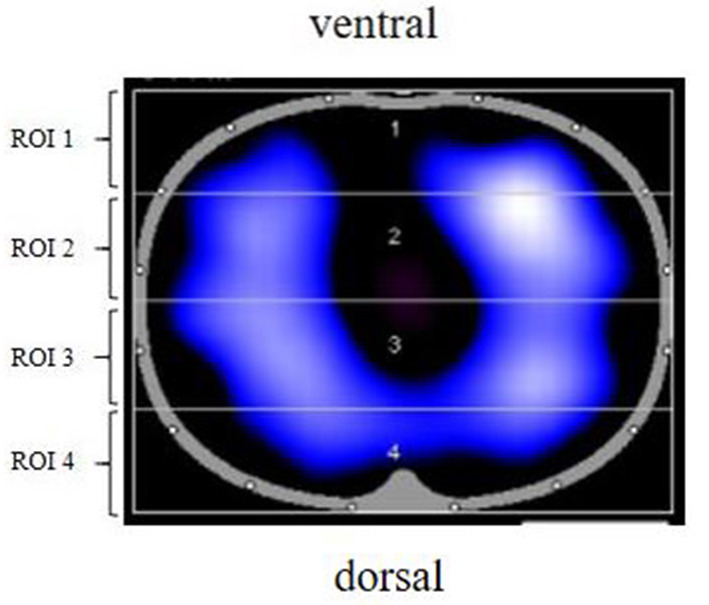
EIT image partition diagram. ROI 1, ROI 2, ROI 3, and ROI 4 were, respectively, from top to bottom. From left to right, right lung and left lung. ROI, region of interest; EIT, electrical impedance tomography.

EIT allows calculation of regional ventilation distribution by assessing change in relative impedance in one predefined cross-sectional slice of the lung. EIT monitoring will be performed using the PulmoVista 500 (Dräger Medical, Lübeck, Germany). A thoracic belt with 16 electrodes will be placed at the fourth intercostal space, such that the 1st electrode will be at the left bound of the sternum and the 16th electrode at the right sternum (Figure 1). EIT images are divided into four equidistant “regions of interest” (ROI) from ventral to dorsal (Figure 2). ROI 1-2 represents the ventral region, namely the non-gravity dependent region. ROI 3 to 4 represents the dorsal region, or gravity-dependent region. Moisture ratio of ROI 1 to 4 can be displayed on the monitor. The electrodes will always be placed in the same mode and kept in place during the entire measurement protocol. Participants will be asked to not speak and to limit trunk movements during the recording period.

### Procedure

Study procedures will be carried out in the following steps:

- Once a patient meets inclusion criteria and provides a written informed consent, the following data will be collected: age, sex, weight, height, BMI, smoking history, type and duration of surgery, comorbidities, arterial blood gas analysis (PH, PaO_2_, PaCO_2_, FiO_2_), chest X-ray or computed tomography (CT), pain (11-point NRS), and the time from admission to the ICU to the first mobilization.
- The following baseline parameters will be recorded when the patient is in decubitus position (T_0_): heart rate (HR), blood pressure (BP), oxygen saturation (SO_2_), respiratory rate (RR), and EIT ROI 1-4. The duration of each EIT recording will be 2 min.
- The patient will be seated in a chair beside the bed with assistance. Oxygen therapy method and oxygen concentration will be the same as that before getting out of bed.
- The parameters presented in step 2 will be recorded at 15 min (T_1_), 30 min (T_2_), 60 min (T_3_), 90 min (T_4_), and 120 min (T_5_) for each patient after getting out of the bed.

After patients reach the 120-min time point, their participation in the study will be complete. No further interaction with the enrolled patients is planned.

### Outcome Measures

The primary outcomes include the improvement in homogeneity of pulmonary ventilation distribution after mobilization and optimal duration of early mobilization. Secondary outcomes include optimal duration of early mobilization for different surgical methods; correlation between bed rest time and optimal duration of early mobilization; and correlation between blood oxygen saturation, oxygenation index, and improvement in homogeneity of pulmonary ventilation distribution.

### Definitions

1. Homogeneity index is defined as the ratio of EIT values of ventral to those of dorsal ROIs; thus, lower levels (~1.0) represent better homogeneity of ventilation (18).
2. Optimal duration of early mobilization is defined as the mobilization time at which homogeneity index is closest to 1.
3. Bed rest time is the time from the start of surgery to the time of first mobilization.

### Adverse Events Management

We do not anticipate any adverse events for patients in this observational study.

### Data Management and Monitoring

Before the initiation of the study, an electronic case report form will be established with password-protected access. Each enrolled patient will be assigned an identification number. Patient data will be coded and kept confidential. We will indicate a coordinator to ensure the integrity of data collection and timely completion of the case report form. Trial conduct will be audited monthly by personnel independent of investigators with no competing interests.

### Statistical Analysis

A prior study that used EIT to assess ventilation distribution in patients undergoing major open abdominal surgery found that a two-sided significance level (α) of 0.05 and a target power of 80% would require 10 subjects (19). We aim to recruit 50 patients, surpassing the minimum necessary for our target power and significance level. Analyses will be conducted using SPSS 21.0 (IBM, Armonk, New York, USA). Continuous variables will be expressed as means (95% confidence intervals, CI) or as medians (interquartile range). Categorical variables will be analyzed using the chi-square test. Means or medians will be compared between groups using the *t*-test, the Mann-Whitney *U*-test, and one-way analysis of variance with F- or Kruskall-Wallis tests, when appropriate. Pearson correlation or Spearman rank correlation coefficient will be used to assess the correlation between variables, as appropriate. Intention-to-treat analyses will be carried out using mixed models. All tests will be 2-sided, and significance will be set at the 0.05 probability level.

### Ethics and Dissemination

The study protocol has been approved by the Institutional Review Board of Dong E Hospital Affiliated to Shandong First Medical University (2020036). The trial is registered at chictr.org.cn with identifier ChiCTR2100042877, registered on January 31, 2021. The results of the study will be presented at relevant national and international conferences and submitted to international peer-reviewed journals. There are no plans to communicate results specifically to participants. Important protocol modifications, such as changes to eligibility criteria, outcomes, or analyses, will be communicated to all relevant parties (including investigators, IRBs, trial participants, trial registries, journals, and regulators) as needed via email or in-person communication.

## Discussion

The purpose of this study is to evaluate the effect of duration of post-operative early mobilization on the distribution of regional ventilation in patients undergoing upper abdominal surgery. Our study will involve the use of EIT that allows for the determination of changes in regional air content corresponding to different mobilization durations based on the changes in electrical impedance caused by inspired gas.

Following a major upper abdominal surgery, episodes of hypoxemia are common, as the restoration of a normal alveolar-to-arterial oxygen difference may take a few days. Functional residual capacity usually reaches its lowest value 1–2 days after surgery before slowly returning to normal values after 5–7 days post-operatively (20–22). Early mobilization prevents PPCs (23); improves pulmonary endurance, muscle strength, and quality of life (24–26); and also provides nursing staff an important measure to integrate the ERAS concept clinically (27). Sitting and standing during the first day after major abdominal surgery increase arterial oxygen saturation (28, 29). In fact, a significant reduction in PPC outcomes (odds ratio 0.08, 95% CI 0.010–0.71) was reported in patients who received enforced mobilization within 24 h after surgery for their gastrointestinal cancer (30). Also, patients undergoing abdominal surgery who were not mobilized (>10 min) within the first 24 h post-operatively were 3 times (95% CI 1.2–8.0) more likely to develop a PPC with each day out of surgery (31). Thus, early mobilization is clinically advised to decrease the risk of PPCs.

Atelectasis seen on CT scans during anesthesia persists for at least 24 h in most patients undergoing a major surgery. Based on a review of post-operative atelectasis in a heterogeneous group of non-thoracic patients, radiological evidence of atelectasis was found in 57% (539/944) of the patients up to 3 days post-operatively, with the diagnosis of atelectasis without radiography particularly challenging in the absence of fever (32). Thus, alternative methods monitoring post-operative lung function are essential to minimize the risk of PPCs (33). The effects of post-operative early mobilization on regional lung ventilation in patients undergoing an upper abdominal surgery have not been studied; EIT can be used clinically to fill this gap.

EIT assesses gas distribution in the lungs by monitoring the changes in thoracic impedance caused by intrapulmonary ventilation; gas distribution thus measured by EIT correlates well with that measured by CT (34–36). In the upright sitting position, the diaphragm is lowered due to the action of gravity, and the area for lung movement is enlarged. This significantly increases the number of alveoli that are effectively involved in breathing, making the distribution of air in the lungs more uniform. Bed rest induces heterogeneity of ventilation, especially in the dorsal regions (37), which can be prevented by modifying positive end-expiratory pressure (PEEP) levels with position changes (38). In this study, the objective is to demonstrate that patient mobilization induces ventilation redistribution without changes in PEEP.

EIT images possess a high temporal and functional resolution, allowing the visualization of dynamic physiological and pathological changes on a breath-by-breath basis. The use of EIT in clinical practice is supported by several studies demonstrating a good correlation between impedance tomography data and other validated methods of measuring lung volume, such as PEEP titration, lung retraction, quantification of pulmonary edema, assessment of mode of ventilation, regional distribution of ventilation, and effects of position changes (39, 40).

Changes in body position are known to influence respiratory mechanics and the pulmonary gas exchange; however, mechanisms involved in the lung aeration and oxygenation improvements are not clear. Nevertheless, we hypothesize that an effective redistribution of air volume induced by the trunk's upright position could prevent respiratory monotony in the resting state and also improve the air volume redistribution (41). Other possible mechanisms include enhanced diaphragm utilization and an increase in transpulmonary pressure. Moreover, because increased tidal impedance variation was observed in the posterior lung regions during active exercise without PaCO_2_ changes, we suggest that oxygenation improvement is not explained by marked increases in minute ventilation. Sitting, standing, and walking increases heart rate and systolic and diastolic blood pressure (42). The active use of a cycle ergometer for 5 min by subjects in the ICU caused a slight increase in the sensation of dyspnea, from mild to moderate and was associated with increases in heart and breathing frequency (43).

### Strengths and Limitations

This is a prospective study designed to demonstrate that early mobilization can improve the distribution of pulmonary ventilation in patients after surgery. The main limitation is that this is a single-center study and may lack external validity. Finally, the EIT measurement is based on a lung slice; therefore, despite a lack of change in the belt position and minimal variation in EIT value induced by different body positions in a healthy population (44), we cannot be certain that the body position change modifies the analyzed lung zone and that the gravity might affect pulmonary tissue or draining of the intrapulmonary liquids in patients with pulmonary illness.

### Study Status

At the time of manuscript submission, the study is in the preparation phase for recruitment. This is the first version of the protocol. Recruitment is scheduled to begin in July 2021 and expected to be completed by June 2022.

## Ethics Statement

The studies involving human participants were reviewed and approved by Institutional Review Board of Dong E Hospital Affiliated to Shandong First Medical University. The patients/participants provided their written informed consent to participate in this study.

## Author Contributions

CW and XL planned the study and critically revised the manuscript. XS performed the design of the study. XS and XL drafted the manuscript. All authors contributed to the design and development of the trial, read, and approved the final manuscript.

## Conflict of Interest

The authors declare that the research was conducted in the absence of any commercial or financial relationships that could be construed as a potential conflict of interest.

## Publisher's Note

All claims expressed in this article are solely those of the authors and do not necessarily represent those of their affiliated organizations, or those of the publisher, the editors and the reviewers. Any product that may be evaluated in this article, or claim that may be made by its manufacturer, is not guaranteed or endorsed by the publisher.
